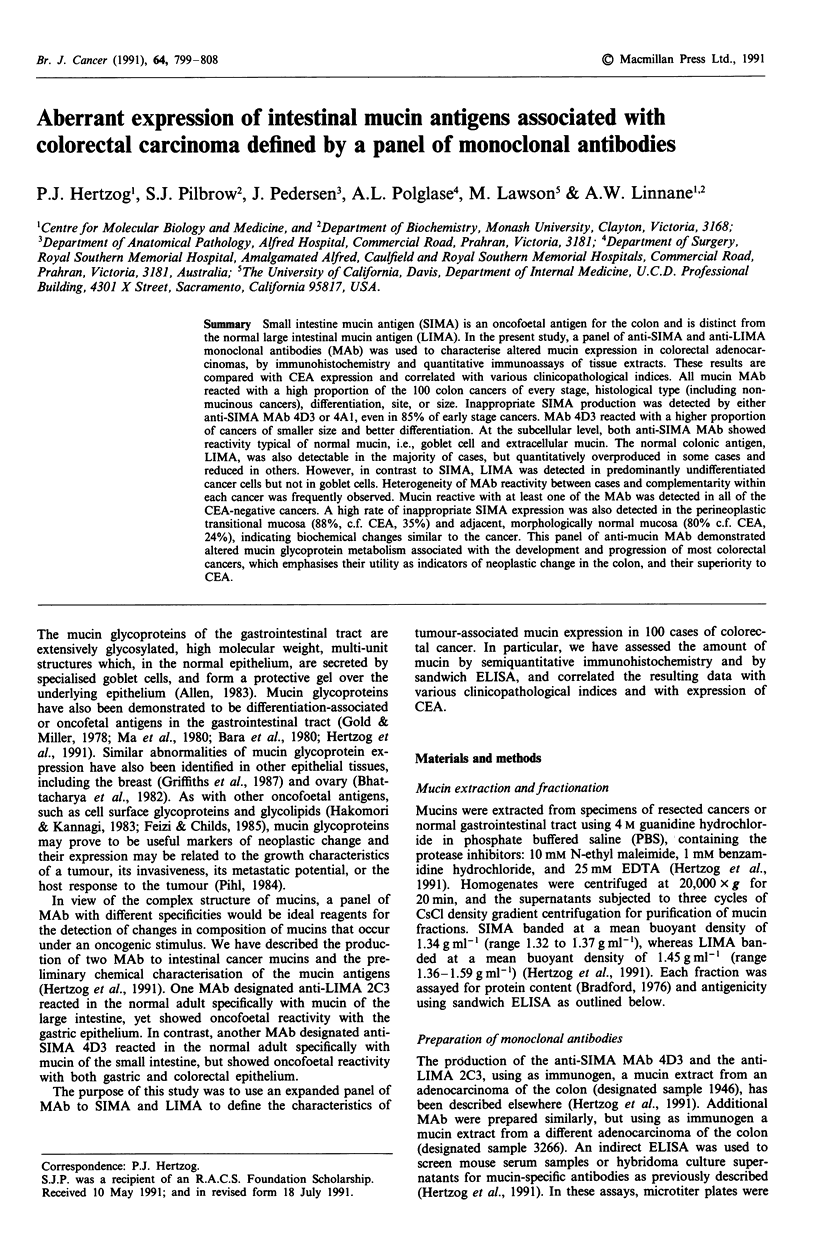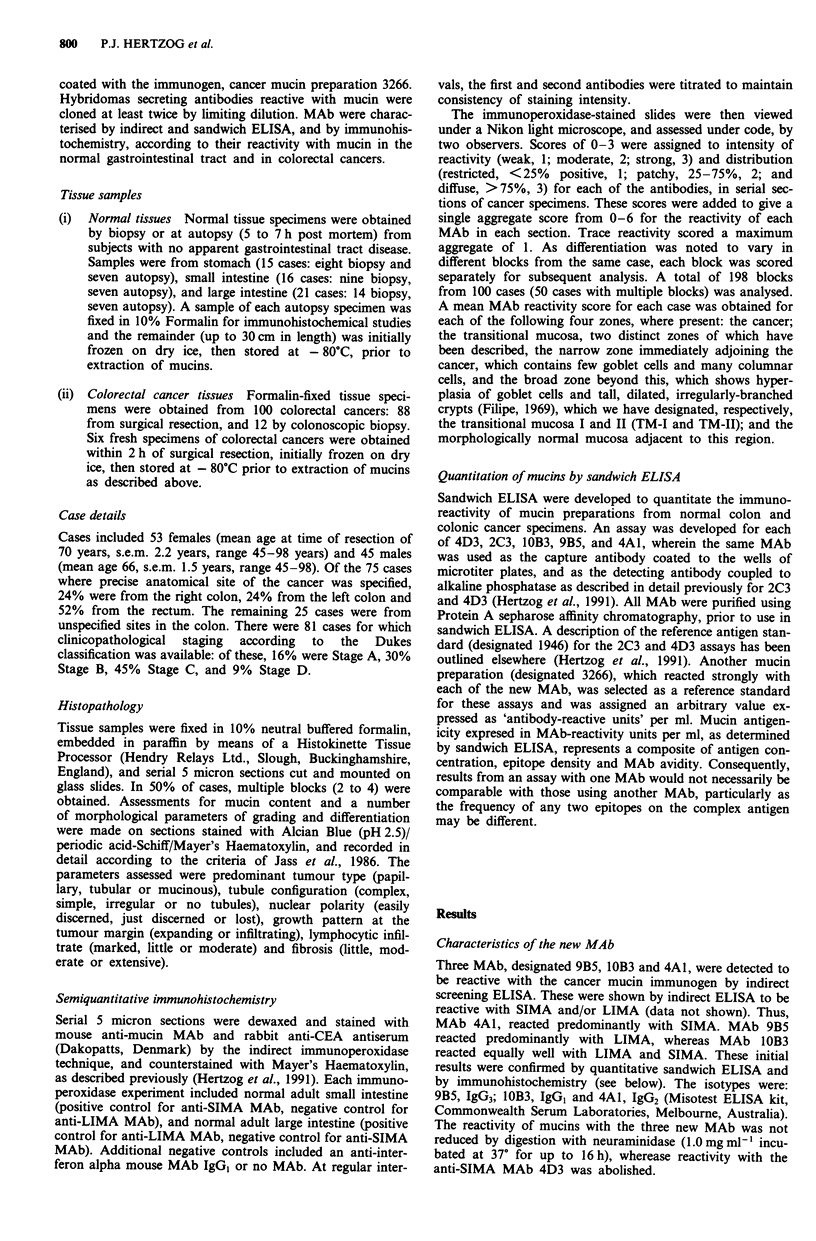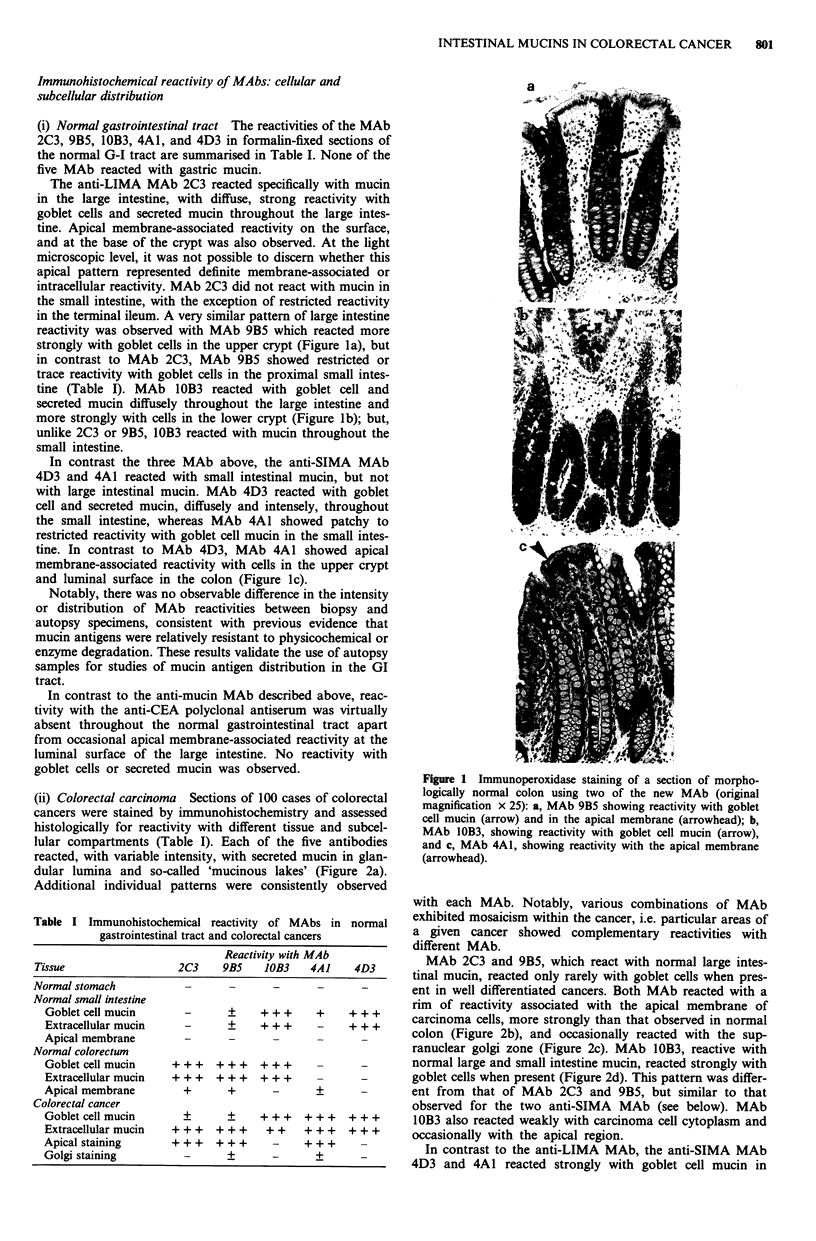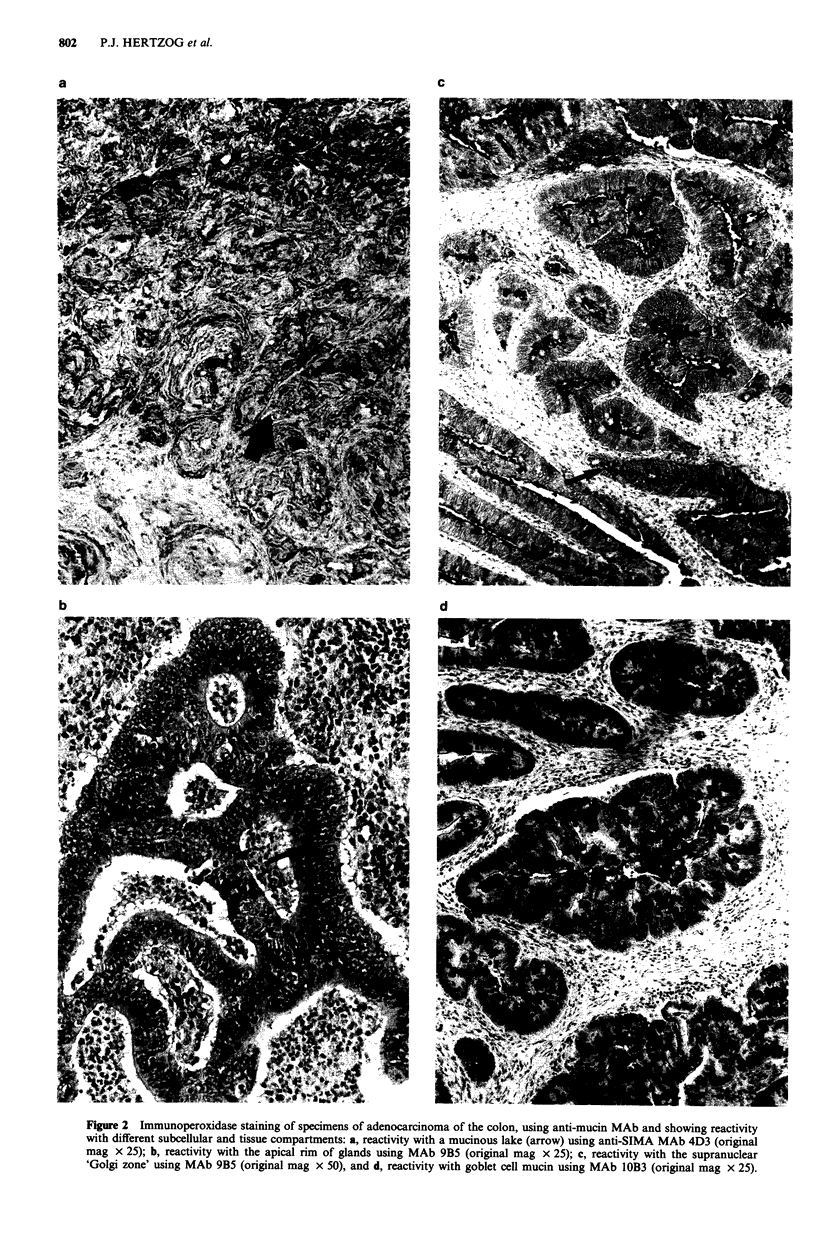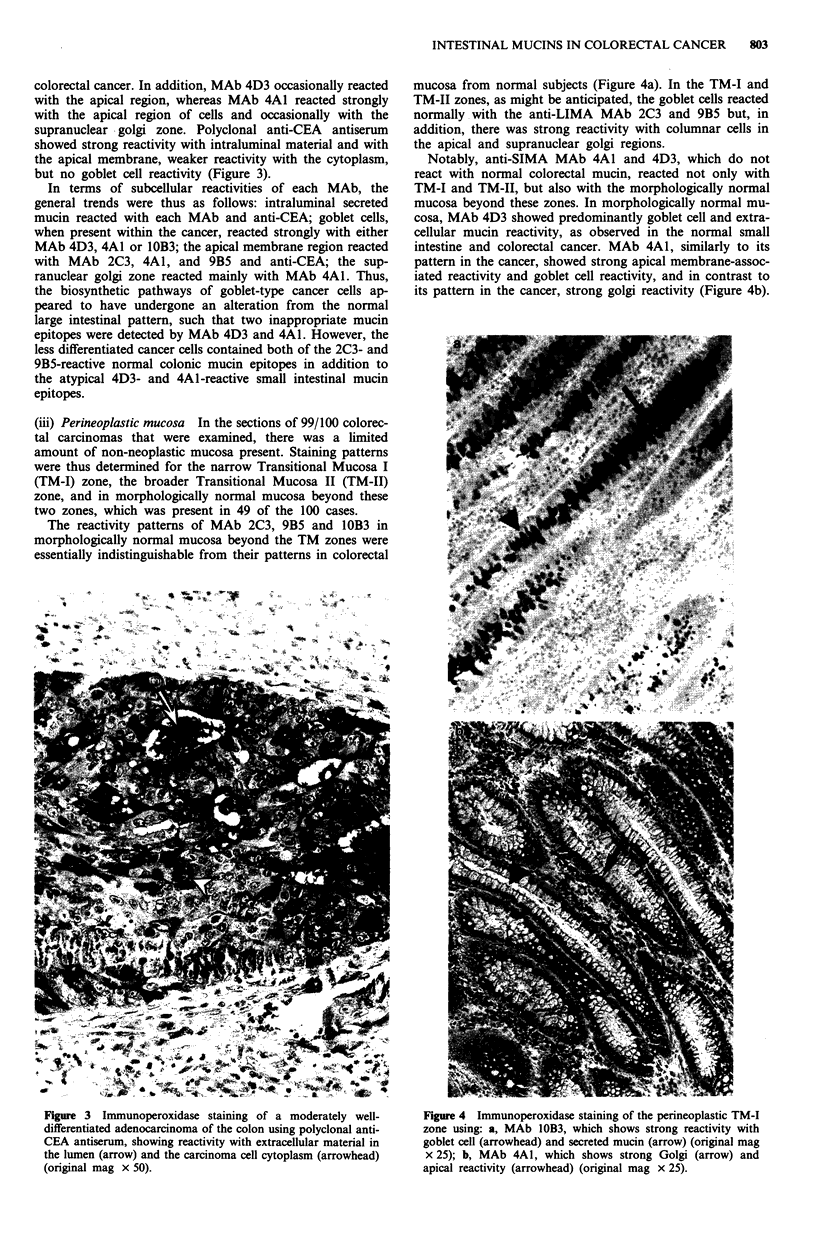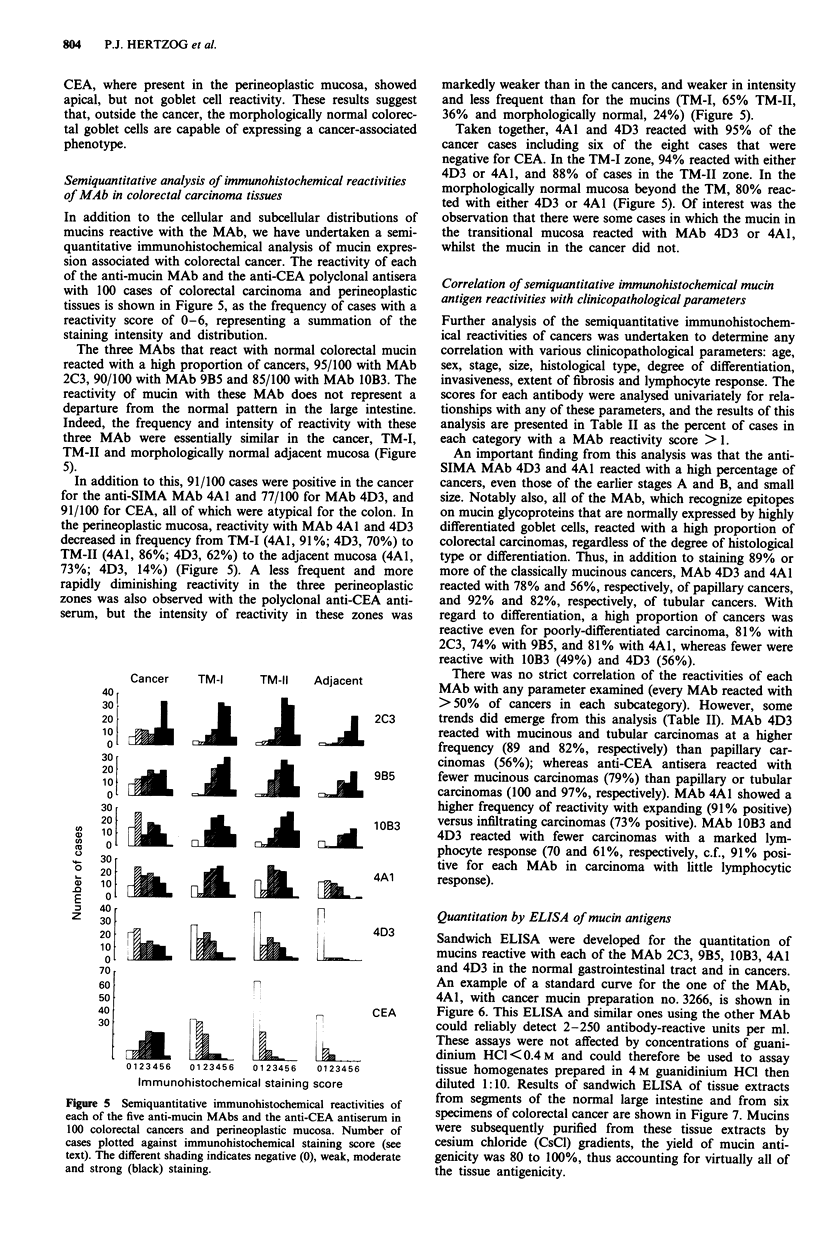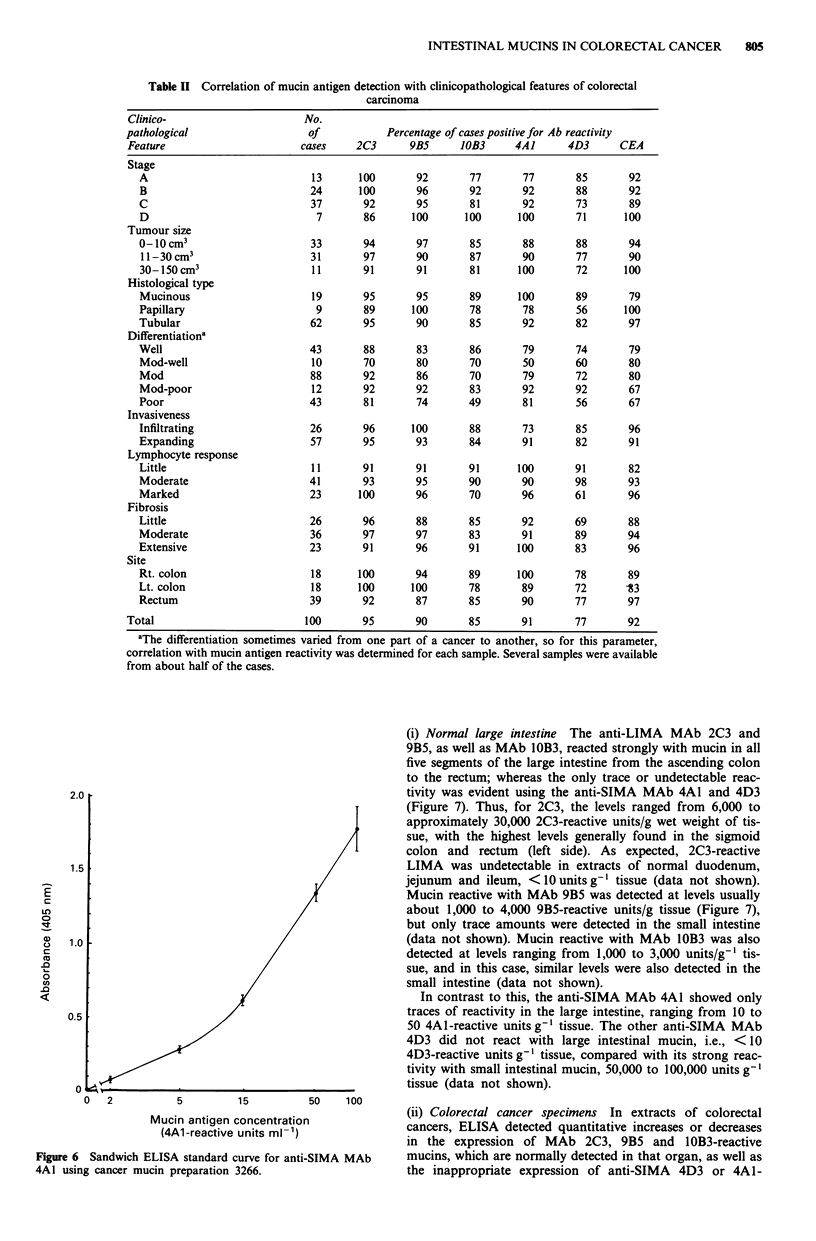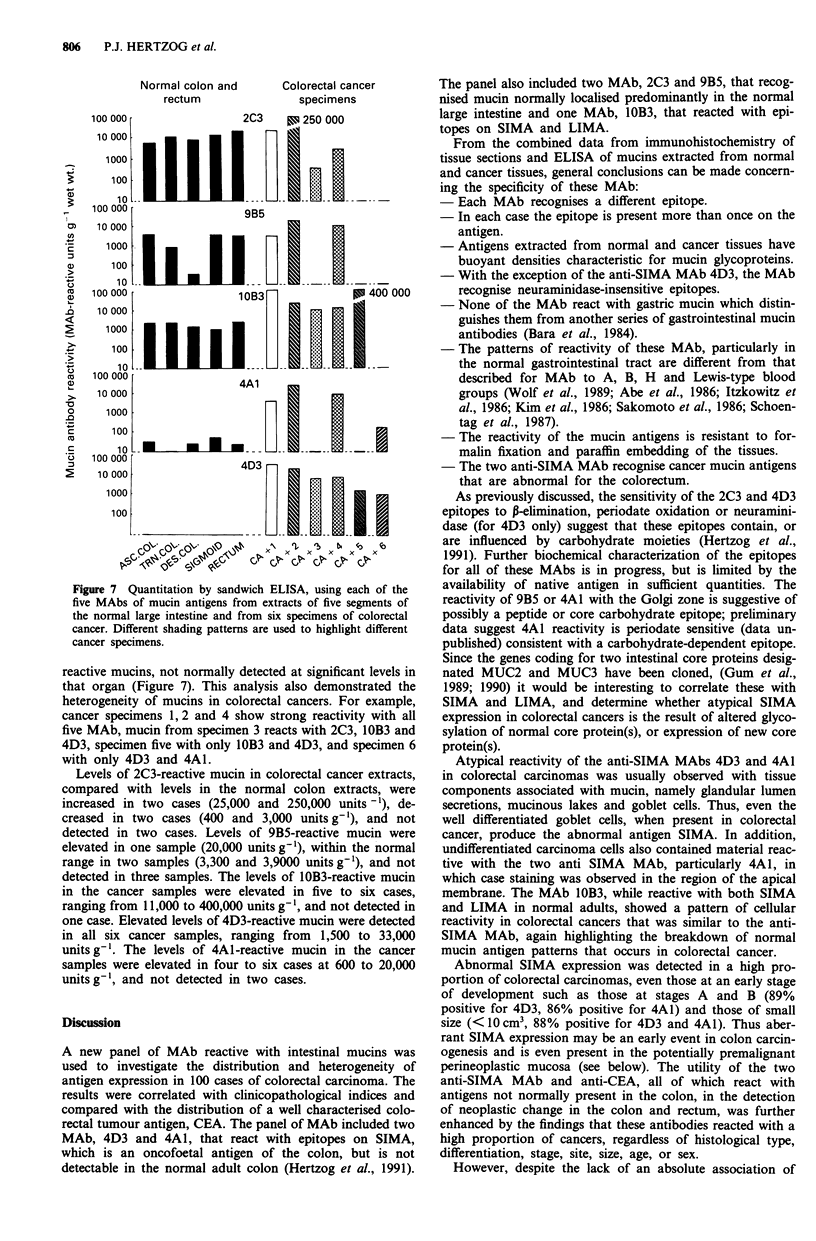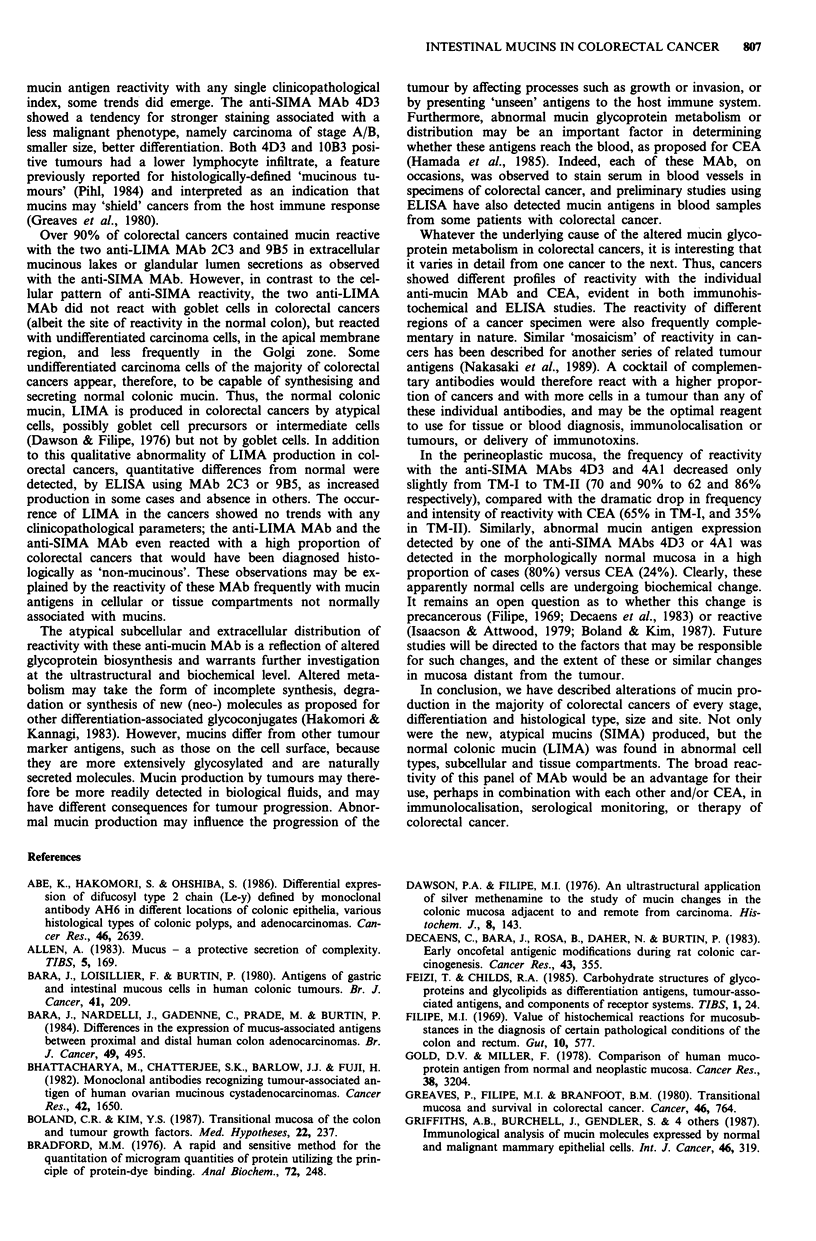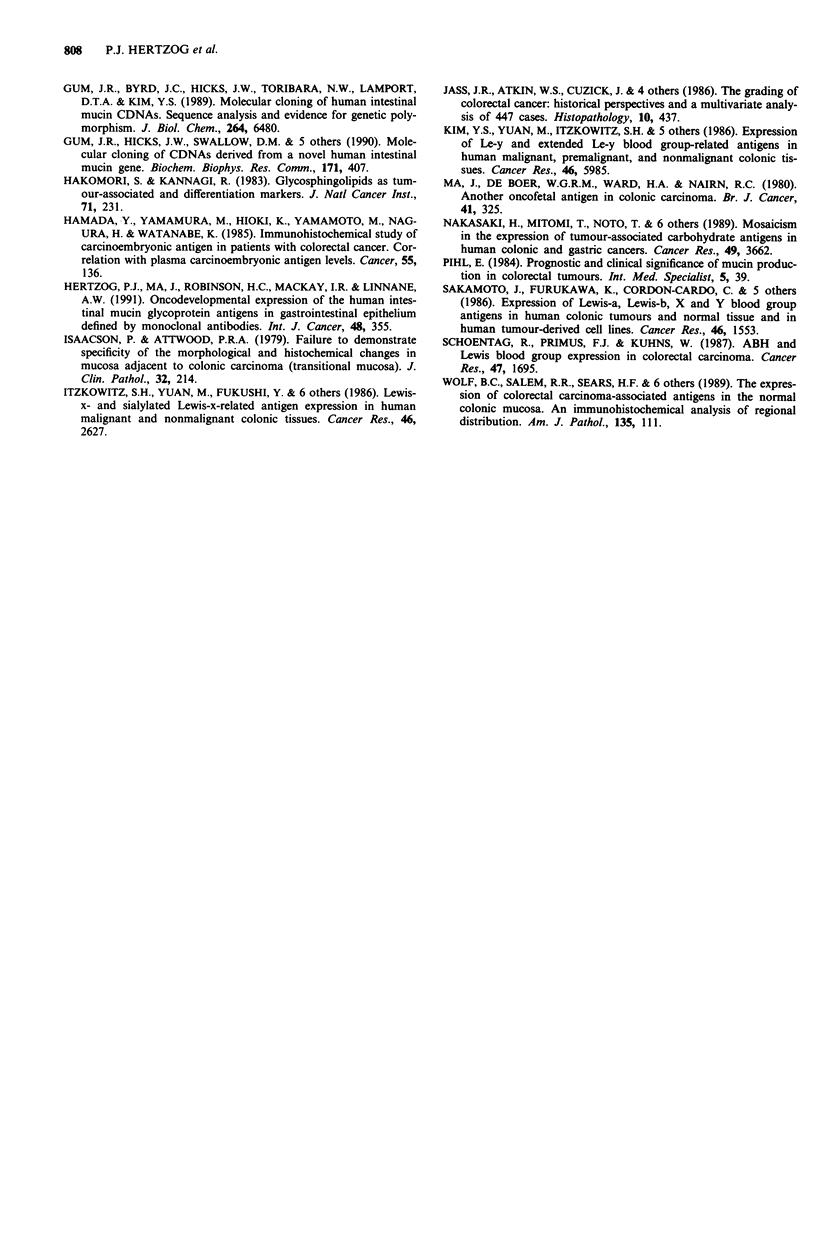# Aberrant expression of intestinal mucin antigens associated with colorectal carcinoma defined by a panel of monoclonal antibodies.

**DOI:** 10.1038/bjc.1991.404

**Published:** 1991-11

**Authors:** P. J. Hertzog, S. J. Pilbrow, J. Pedersen, A. L. Polglase, M. Lawson, A. W. Linnane

**Affiliations:** Centre for Molecular Biology and Medicine, Monash University, Clayton, Victoria, Australia.

## Abstract

**Images:**


*Br. J. Cancer* (1991), **64**, 799–808 © Macmillan Press Ltd., 1991

### Aberrant expression of intestinal mucin antigens associated with colorectal carcinoma defined by a panel of monoclonal antibodies

P.J. Hertzog[1], S.J. Pilbrow[2], J. Pedersen[3], A.L. Polglase[4], M. Lawson[5] & A.W. Linnane[1,2]

[1]*Centre for Molecular Biology and Medicine, and* [2]*Department of Biochemistry, Monash University, Clayton, Victoria, 3168;* [3]*Department of Anatomical Pathology, Alfred Hospital, Commercial Road, Prahran, Victoria, 3181;* [4]*Department of Surgery, Royal Southern Memorial Hospital, Amalgamated Alfred, Caulfield and Royal Southern Memorial Hospitals, Commercial Road, Prahran, Victoria, 3181, Australia;* [5]*The University of California, Davis, Department of Internal Medicine, U.C.D. Professional Building, 4301 X Street, Sacramento, California 95817, USA.*

**Summary** Small intestine mucin antigen (SIMA) is an oncofoetal antigen for the colon and is distinct from the normal large intestinal mucin antigen (LIMA). In the present study, a panel of anti-SIMA and anti-LIMA monoclonal antibodies (MAb) was used to characterise altered mucin expression in colorectal adenocarcinomas, by immunohistochemistry and quantitative immunoassays of tissue extracts. These results are compared with CEA expression and correlated with various clinicopathological indices. All mucin MAb reacted with a high proportion of the 100 colon cancers of every stage, histological type (including non-mucinous cancers), differentiation, site, or size. Inappropriate SIMA production was detected by either anti-SIMA MAb 4D3 or 4A1, even in 85% of early stage cancers. MAb 4D3 reacted with a higher proportion of cancers of smaller size and better differentiation. At the subcellular level, both anti-SIMA MAb showed reactivity typical of normal mucin, i.e., goblet cell and extracellular mucin. The normal colonic antigen, LIMA, was also detectable in the majority of cases, but quantitatively overproduced in some cases and reduced in others. However, in contrast to SIMA, LIMA was detected in predominantly undifferentiated cancer cells but not in goblet cells. Heterogeneity of MAb reactivity between cases and complementarity within each cancer was frequently observed. Mucin reactive with at least one of the MAb was detected in all of the CEA-negative cancers. A high rate of inappropriate SIMA expression was also detected in the perineoplastic transitional mucosa (88%, c.f. CEA, 35%) and adjacent, morphologically normal mucosa (80% c.f. CEA, 24%), indicating biochemical changes similar to the cancer. This panel of anti-mucin MAb demonstrated altered mucin glycoprotein metabolism associated with the development and progression of most colorectal cancers, which emphasises their utility as indicators of neoplastic change in the colon, and their superiority to CEA.

The mucin glycoproteins of the gastrointestinal tract are extensively glycosylated, high molecular weight, multi-unit structures which, in the normal epithelium, are secreted by specialised goblet cells, and form a protective gel over the underlying epithelium (Allen, 1983). Mucin glycoproteins have also been demonstrated to be differentiation-associated or oncofetal antigens in the gastrointestinal tract (Gold & Miller, 1978; Ma *et al.*, 1980; Bara *et al.*, 1980; Hertzog *et al.*, 1991). Similar abnormalities of mucin glycoprotein expression have also been identified in other epithelial tissues, including the breast (Griffiths *et al.*, 1987) and ovary (Bhattacharya *et al.*, 1982). As with other oncofoetal antigens, such as cell surface glycoproteins and glycolipids (Hakomori & Kannagi, 1983; Feizi & Childs, 1985), mucin glycoproteins may prove to be useful markers of neoplastic change and their expression may be related to the growth characteristics of a tumour, its invasiveness, its metastatic potential, or the host response to the tumour (Pihl, 1984).

In view of the complex structure of mucins, a panel of MAb with different specificities would be ideal reagents for the detection of changes in composition of mucins that occur under an oncogenic stimulus. We have described the production of two MAb to intestinal cancer mucins and the preliminary chemical characterisation of the mucin antigens (Hertzog *et al.*, 1991). One MAb designated anti-LIMA 2C3 reacted in the normal adult specifically with mucin of the large intestine, yet showed oncofoetal reactivity with the gastric epithelium. In contrast, another MAb designated anti-SIMA 4D3 reacted in the normal adult specifically with mucin of the small intestine, but showed oncofoetal reactivity with both gastric and colorectal epithelium.

The purpose of this study was to use an expanded panel of MAb to SIMA and LIMA to define the characteristics of tumour-associated mucin expression in 100 cases of colorectal cancer. In particular, we have assessed the amount of mucin by semiquantitative immunohistochemistry and by sandwich ELISA, and correlated the resulting data with various clinicopathological indices and with expression of CEA.

### Materials and methods

### *Mucin extraction and fractionation*

Mucins were extracted from specimens of resected cancers or normal gastrointestinal tract using 4 M guanidine hydrochloride in phosphate buffered saline (PBS), containing the protease inhibitors: 10 mM N-ethyl maleimide, 1 mM benzamidine hydrochloride, and 25 mM EDTA (Hertzog *et al.*, 1991). Homogenates were centrifuged at $20,000 \times g$ for 20 min, and the supernatants subjected to three cycles of CsCl density gradient centrifugation for purification of mucin fractions. SIMA banded at a mean buoyant density of $1.34\ g\ ml^{-1}$ (range 1.32 to $1.37\ g\ ml^{-1}$), whereas LIMA banded at a mean buoyant density of $1.45\ g\ ml^{-1}$ (range $1.36-1.59\ g\ ml^{-1}$) (Hertzog *et al.*, 1991). Each fraction was assayed for protein content (Bradford, 1976) and antigenicity using sandwich ELISA as outlined below.

### *Preparation of monoclonal antibodies*

The production of the anti-SIMA MAb 4D3 and the anti-LIMA 2C3, using as immunogen, a mucin extract from an adenocarcinoma of the colon (designated sample 1946), has been described elsewhere (Hertzog *et al.*, 1991). Additional MAb were prepared similarly, but using as immunogen a mucin extract from a different adenocarcinoma of the colon (designated sample 3266). An indirect ELISA was used to screen mouse serum samples or hybridoma culture supernatants for mucin-specific antibodies as previously described (Hertzog *et al.*, 1991). In these assays, microtiter plates were

Correspondence: P.J. Hertzog.
S.J.P. was a recipient of an R.A.C.S. Foundation Scholarship.
Received 10 May 1991; and in revised form 18 July 1991.

coated with the immunogen, cancer mucin preparation 3266. Hybridomas secreting antibodies reactive with mucin were cloned at least twice by limiting dilution. MAb were characterised by indirect and sandwich ELISA, and by immunohistochemistry, according to their reactivity with mucin in the normal gastrointestinal tract and in colorectal cancers.

### Tissue samples

(i) *Normal tissues* Normal tissue specimens were obtained by biopsy or at autopsy (5 to 7 h post mortem) from subjects with no apparent gastrointestinal tract disease. Samples were from stomach (15 cases: eight biopsy and seven autopsy), small intestine (16 cases: nine biopsy, seven autopsy), and large intestine (21 cases: 14 biopsy, seven autopsy). A sample of each autopsy specimen was fixed in 10% Formalin for immunohistochemical studies and the remainder (up to 30 cm in length) was initially frozen on dry ice, then stored at − 80°C, prior to extraction of mucins.

(ii) *Colorectal cancer tissues* Formalin-fixed tissue specimens were obtained from 100 colorectal cancers: 88 from surgical resection, and 12 by colonoscopic biopsy. Six fresh specimens of colorectal cancers were obtained within 2 h of surgical resection, initially frozen on dry ice, then stored at − 80°C prior to extraction of mucins as described above.

### Case details

Cases included 53 females (mean age at time of resection of 70 years, s.e.m. 2.2 years, range 45–98 years) and 45 males (mean age 66, s.e.m. 1.5 years, range 45–98). Of the 75 cases where precise anatomical site of the cancer was specified, 24% were from the right colon, 24% from the left colon and 52% from the rectum. The remaining 25 cases were from unspecified sites in the colon. There were 81 cases for which clinicopathological staging according to the Dukes classification was available: of these, 16% were Stage A, 30% Stage B, 45% Stage C, and 9% Stage D.

### Histopathology

Tissue samples were fixed in 10% neutral buffered formalin, embedded in paraffin by means of a Histokinette Tissue Processor (Hendry Relays Ltd., Slough, Buckinghamshire, England), and serial 5 micron sections cut and mounted on glass slides. In 50% of cases, multiple blocks (2 to 4) were obtained. Assessments for mucin content and a number of morphological parameters of grading and differentiation were made on sections stained with Alcian Blue (pH 2.5)/periodic acid-Schiff/Mayer's Haematoxylin, and recorded in detail according to the criteria of Jass *et al.*, 1986. The parameters assessed were predominant tumour type (papillary, tubular or mucinous), tubule configuration (complex, simple, irregular or no tubules), nuclear polarity (easily discerned, just discerned or lost), growth pattern at the tumour margin (expanding or infiltrating), lymphocytic infiltrate (marked, little or moderate) and fibrosis (little, moderate or extensive).

### Semiquantitative immunohistochemistry

Serial 5 micron sections were dewaxed and stained with mouse anti-mucin MAb and rabbit anti-CEA antiserum (Dakopatts, Denmark) by the indirect immunoperoxidase technique, and counterstained with Mayer's Haematoxylin, as described previously (Hertzog *et al.*, 1991). Each immunoperoxidase experiment included normal adult small intestine (positive control for anti-SIMA MAb, negative control for anti-LIMA MAb), and normal adult large intestine (positive control for anti-LIMA MAb, negative control for anti-SIMA MAb). Additional negative controls included an anti-interferon alpha mouse MAb $IgG_1$ or no MAb. At regular intervals, the first and second antibodies were titrated to maintain consistency of staining intensity.

The immunoperoxidase-stained slides were then viewed under a Nikon light microscope, and assessed under code, by two observers. Scores of 0–3 were assigned to intensity of reactivity (weak, 1; moderate, 2; strong, 3) and distribution (restricted, <25% positive, 1; patchy, 25–75%, 2; and diffuse, >75%, 3) for each of the antibodies, in serial sections of cancer specimens. These scores were added to give a single aggregate score from 0–6 for the reactivity of each MAb in each section. Trace reactivity scored a maximum aggregate of 1. As differentiation was noted to vary in different blocks from the same case, each block was scored separately for subsequent analysis. A total of 198 blocks from 100 cases (50 cases with multiple blocks) was analysed. A mean MAb reactivity score for each case was obtained for each of the following four zones, where present: the cancer; the transitional mucosa, two distinct zones of which have been described, the narrow zone immediately adjoining the cancer, which contains few goblet cells and many columnar cells, and the broad zone beyond this, which shows hyperplasia of goblet cells and tall, dilated, irregularly-branched crypts (Filipe, 1969), which we have designated, respectively, the transitional mucosa I and II (TM-I and TM-II); and the morphologically normal mucosa adjacent to this region.

### Quantitation of mucins by sandwich ELISA

Sandwich ELISA were developed to quantitate the immunoreactivity of mucin preparations from normal colon and colonic cancer specimens. An assay was developed for each of 4D3, 2C3, 10B3, 9B5, and 4A1, wherein the same MAb was used as the capture antibody coated to the wells of microtiter plates, and as the detecting antibody coupled to alkaline phosphatase as described in detail previously for 2C3 and 4D3 (Hertzog *et al.*, 1991). All MAb were purified using Protein A sepharose affinity chromatography, prior to use in sandwich ELISA. A description of the reference antigen standard (designated 1946) for the 2C3 and 4D3 assays has been outlined elsewhere (Hertzog *et al.*, 1991). Another mucin preparation (designated 3266), which reacted strongly with each of the new MAb, was selected as a reference standard for these assays and was assigned an arbitrary value expressed as 'antibody-reactive units' per ml. Mucin antigenicity expressed in MAb-reactivity units per ml, as determined by sandwich ELISA, represents a composite of antigen concentration, epitope density and MAb avidity. Consequently, results from an assay with one MAb would not necessarily be comparable with those using another MAb, particularly as the frequency of any two epitopes on the complex antigen may be different.

### Results

### Characteristics of the new MAb

Three MAb, designated 9B5, 10B3 and 4A1, were detected to be reactive with the cancer mucin immunogen by indirect screening ELISA. These were shown by indirect ELISA to be reactive with SIMA and/or LIMA (data not shown). Thus, MAb 4A1, reacted predominantly with SIMA. MAb 9B5 reacted predominantly with LIMA, whereas MAb 10B3 reacted equally well with LIMA and SIMA. These initial results were confirmed by quantitative sandwich ELISA and by immunohistochemistry (see below). The isotypes were: 9B5, $IgG_3$; 10B3, $IgG_1$ and 4A1, $IgG_2$ (Misotest ELISA kit, Commonwealth Serum Laboratories, Melbourne, Australia). The reactivity of mucins with the three new MAb was not reduced by digestion with neuraminidase (1.0 mg $ml^{-1}$ incubated at 37° for up to 16 h), wherease reactivity with the anti-SIMA MAb 4D3 was abolished.

*Immunohistochemical reactivity of MAbs: cellular and subcellular distribution*

(i) *Normal gastrointestinal tract* The reactivities of the MAb 2C3, 9B5, 10B3, 4A1, and 4D3 in formalin-fixed sections of the normal G-I tract are summarised in Table I. None of the five MAb reacted with gastric mucin.

The anti-LIMA MAb 2C3 reacted specifically with mucin in the large intestine, with diffuse, strong reactivity with goblet cells and secreted mucin throughout the large intestine. Apical membrane-associated reactivity on the surface, and at the base of the crypt was also observed. At the light microscopic level, it was not possible to discern whether this apical pattern represented definite membrane-associated or intracellular reactivity. MAb 2C3 did not react with mucin in the small intestine, with the exception of restricted reactivity in the terminal ileum. A very similar pattern of large intestine reactivity was observed with MAb 9B5 which reacted more strongly with goblet cells in the upper crypt (Figure 1a), but in contrast to MAb 2C3, MAb 9B5 showed restricted or trace reactivity with goblet cells in the proximal small intestine (Table I). MAb 10B3 reacted with goblet cell and secreted mucin diffusely throughout the large intestine and more strongly with cells in the lower crypt (Figure 1b); but, unlike 2C3 or 9B5, 10B3 reacted with mucin throughout the small intestine.

In contrast the three MAb above, the anti-SIMA MAb 4D3 and 4A1 reacted with small intestinal mucin, but not with large intestinal mucin. MAb 4D3 reacted with goblet cell and secreted mucin, diffusely and intensely, throughout the small intestine, whereas MAb 4A1 showed patchy to restricted reactivity with goblet cell mucin in the small intestine. In contrast to MAb 4D3, MAb 4A1 showed apical membrane-associated reactivity with cells in the upper crypt and luminal surface in the colon (Figure 1c).

Notably, there was no observable difference in the intensity or distribution of MAb reactivities between biopsy and autopsy specimens, consistent with previous evidence that mucin antigens were relatively resistant to physicochemical or enzyme degradation. These results validate the use of autopsy samples for studies of mucin antigen distribution in the GI tract.

In contrast to the anti-mucin MAb described above, reactivity with the anti-CEA polyclonal antiserum was virtually absent throughout the normal gastrointestinal tract apart from occasional apical membrane-associated reactivity at the luminal surface of the large intestine. No reactivity with goblet cells or secreted mucin was observed.

(ii) *Colorectal carcinoma* Sections of 100 cases of colorectal cancers were stained by immunohistochemistry and assessed histologically for reactivity with different tissue and subcellular compartments (Table I). Each of the five antibodies reacted, with variable intensity, with secreted mucin in glandular lumina and so-called 'mucinous lakes' (Figure 2a). Additional individual patterns were consistently observed

**Table I** Immunohistochemical reactivity of MAbs in normal gastrointestinal tract and colorectal cancers

| | *Reactivity with MAb* | | | | |
|---|---|---|---|---|---|
| *Tissue* | *2C3* | *9B5* | *10B3* | *4A1* | *4D3* |
| *Normal stomach* | – | – | – | – | – |
| *Normal small intestine* | | | | | |
| Goblet cell mucin | – | ± | +++ | + | +++ |
| Extracellular mucin | – | ± | +++ | – | +++ |
| Apical membrane | – | – | – | – | – |
| *Normal colorectum* | | | | | |
| Goblet cell mucin | +++ | +++ | +++ | – | – |
| Extracellular mucin | +++ | +++ | +++ | – | – |
| Apical membrane | + | + | – | ± | – |
| *Colorectal cancer* | | | | | |
| Goblet cell mucin | ± | ± | +++ | +++ | +++ |
| Extracellular mucin | +++ | +++ | ++ | +++ | +++ |
| Apical staining | +++ | +++ | – | +++ | – |
| Golgi staining | – | ± | – | ± | – |

**Figure 1** Immunoperoxidase staining of a section of morphologically normal colon using two of the new MAb (original magnification × 25): **a**, MAb 9B5 showing reactivity with goblet cell mucin (arrow) and in the apical membrane (arrowhead); **b**, MAb 10B3, showing reactivity with goblet cell mucin (arrow), and **c**, MAb 4A1, showing reactivity with the apical membrane (arrowhead).

with each MAb. Notably, various combinations of MAb exhibited mosaicism within the cancer, i.e. particular areas of a given cancer showed complementary reactivities with different MAb.

MAb 2C3 and 9B5, which react with normal large intestinal mucin, reacted only rarely with goblet cells when present in well differentiated cancers. Both MAb reacted with a rim of reactivity associated with the apical membrane of carcinoma cells, more strongly than that observed in normal colon (Figure 2b), and occasionally reacted with the supranuclear golgi zone (Figure 2c). MAb 10B3, reactive with normal large and small intestine mucin, reacted strongly with goblet cells when present (Figure 2d). This pattern was different from that of MAb 2C3 and 9B5, but similar to that observed for the two anti-SIMA MAb (see below). MAb 10B3 also reacted weakly with carcinoma cell cytoplasm and occasionally with the apical region.

In contrast to the anti-LIMA MAb, the anti-SIMA MAb 4D3 and 4A1 reacted strongly with goblet cell mucin in

**Figure 2** Immunoperoxidase staining of specimens of adenocarcinoma of the colon, using anti-mucin MAb and showing reactivity with different subcellular and tissue compartments: **a**, reactivity with a mucinous lake (arrow) using anti-SIMA MAb 4D3 (original mag × 25); **b**, reactivity with the apical rim of glands using MAb 9B5 (original mag × 25); **c**, reactivity with the supranuclear 'Golgi zone' using MAb 9B5 (original mag × 50), and **d**, reactivity with goblet cell mucin using MAb 10B3 (original mag × 25).

colorectal cancer. In addition, MAb 4D3 occasionally reacted with the apical region, whereas MAb 4A1 reacted strongly with the apical region of cells and occasionally with the supranuclear golgi zone. Polyclonal anti-CEA antiserum showed strong reactivity with intraluminal material and with the apical membrane, weaker reactivity with the cytoplasm, but no goblet cell reactivity (Figure 3).

In terms of subcellular reactivities of each MAb, the general trends were thus as follows: intraluminal secreted mucin reacted with each MAb and anti-CEA; goblet cells, when present within the cancer, reacted strongly with either MAb 4D3, 4A1 or 10B3; the apical membrane region reacted with MAb 2C3, 4A1, and 9B5 and anti-CEA; the supranuclear golgi zone reacted mainly with MAb 4A1. Thus, the biosynthetic pathways of goblet-type cancer cells appeared to have undergone an alteration from the normal large intestinal pattern, such that two inappropriate mucin epitopes were detected by MAb 4D3 and 4A1. However, the less differentiated cancer cells contained both of the 2C3- and 9B5-reactive normal colonic mucin epitopes in addition to the atypical 4D3- and 4A1-reactive small intestinal mucin epitopes.

(iii) *Perineoplastic mucosa* In the sections of 99/100 colorectal carcinomas that were examined, there was a limited amount of non-neoplastic mucosa present. Staining patterns were thus determined for the narrow Transitional Mucosa I (TM-I) zone, the broader Transitional Mucosa II (TM-II) zone, and in morphologically normal mucosa beyond these two zones, which was present in 49 of the 100 cases.

The reactivity patterns of MAb 2C3, 9B5 and 10B3 in morphologically normal mucosa beyond the TM zones were essentially indistinguishable from their patterns in colorectal mucosa from normal subjects (Figure 4a). In the TM-I and TM-II zones, as might be anticipated, the goblet cells reacted normally with the anti-LIMA MAb 2C3 and 9B5 but, in addition, there was strong reactivity with columnar cells in the apical and supranuclear golgi regions.

Notably, anti-SIMA MAb 4A1 and 4D3, which do not react with normal colorectal mucin, reacted not only with TM-I and TM-II, but also with the morphologically normal mucosa beyond these zones. In morphologically normal mucosa, MAb 4D3 showed predominantly goblet cell and extracellular mucin reactivity, as observed in the normal small intestine and colorectal cancer. MAb 4A1, similarly to its pattern in the cancer, showed strong apical membrane-associated reactivity and goblet cell reactivity, and in contrast to its pattern in the cancer, strong golgi reactivity (Figure 4b).

**Figure 3** Immunoperoxidase staining of a moderately well-differentiated adenocarcinoma of the colon using polyclonal anti-CEA antiserum, showing reactivity with extracellular material in the lumen (arrow) and the carcinoma cell cytoplasm (arrowhead) (original mag × 50).

**Figure 4** Immunoperoxidase staining of the perineoplastic TM-I zone using: **a**, MAb 10B3, which shows strong reactivity with goblet cell (arrowhead) and secreted mucin (arrow) (original mag × 25); **b**, MAb 4A1, which shows strong Golgi (arrow) and apical reactivity (arrowhead) (original mag × 25).

CEA, where present in the perineoplastic mucosa, showed apical, but not goblet cell reactivity. These results suggest that, outside the cancer, the morphologically normal colorectal goblet cells are capable of expressing a cancer-associated phenotype.

*Semiquantitative analysis of immunohistochemical reactivities of MAb in colorectal carcinoma tissues*

In addition to the cellular and subcellular distributions of mucins reactive with the MAb, we have undertaken a semiquantitative immunohistochemical analysis of mucin expression associated with colorectal cancer. The reactivity of each of the anti-mucin MAb and the anti-CEA polyclonal antisera with 100 cases of colorectal carcinoma and perineoplastic tissues is shown in Figure 5, as the frequency of cases with a reactivity score of 0–6, representing a summation of the staining intensity and distribution.

The three MAbs that react with normal colorectal mucin reacted with a high proportion of cancers, 95/100 with MAb 2C3, 90/100 with MAb 9B5 and 85/100 with MAb 10B3. The reactivity of mucin with these MAb does not represent a departure from the normal pattern in the large intestine. Indeed, the frequency and intensity of reactivity with these three MAb were essentially similar in the cancer, TM-I, TM-II and morphologically normal adjacent mucosa (Figure 5).

In addition to this, 91/100 cases were positive in the cancer for the anti-SIMA MAb 4A1 and 77/100 for MAb 4D3, and 91/100 for CEA, all of which were atypical for the colon. In the perineoplastic mucosa, reactivity with MAb 4A1 and 4D3 decreased in frequency from TM-I (4A1, 91%; 4D3, 70%) to TM-II (4A1, 86%; 4D3, 62%) to the adjacent mucosa (4A1, 73%; 4D3, 14%) (Figure 5). A less frequent and more rapidly diminishing reactivity in the three perineoplastic zones was also observed with the polyclonal anti-CEA antiserum, but the intensity of reactivity in these zones was

**Figure 5** Semiquantitative immunohistochemical reactivities of each of the five anti-mucin MAbs and the anti-CEA antiserum in 100 colorectal cancers and perineoplastic mucosa. Number of cases plotted against immunohistochemical staining score (see text). The different shading indicates negative (0), weak, moderate and strong (black) staining.

markedly weaker than in the cancers, and weaker in intensity and less frequent than for the mucins (TM-I, 65% TM-II, 36% and morphologically normal, 24%) (Figure 5).

Taken together, 4A1 and 4D3 reacted with 95% of the cancer cases including six of the eight cases that were negative for CEA. In the TM-I zone, 94% reacted with either 4D3 or 4A1, and 88% of cases in the TM-II zone. In the morphologically normal mucosa beyond the TM, 80% reacted with either 4D3 or 4A1 (Figure 5). Of interest was the observation that there were some cases in which the mucin in the transitional mucosa reacted with MAb 4D3 or 4A1, whilst the mucin in the cancer did not.

*Correlation of semiquantitative immunohistochemical mucin antigen reactivities with clinicopathological parameters*

Further analysis of the semiquantitative immunohistochemical reactivities of cancers was undertaken to determine any correlation with various clinicopathological parameters: age, sex, stage, size, histological type, degree of differentiation, invasiveness, extent of fibrosis and lymphocyte response. The scores for each antibody were analysed univariately for relationships with any of these parameters, and the results of this analysis are presented in Table II as the percent of cases in each category with a MAb reactivity score $>1$.

An important finding from this analysis was that the anti-SIMA MAb 4D3 and 4A1 reacted with a high percentage of cancers, even those of the earlier stages A and B, and small size. Notably also, all of the MAb, which recognize epitopes on mucin glycoproteins that are normally expressed by highly differentiated goblet cells, reacted with a high proportion of colorectal carcinomas, regardless of the degree of histological type or differentiation. Thus, in addition to staining 89% or more of the classically mucinous cancers, MAb 4D3 and 4A1 reacted with 78% and 56%, respectively, of papillary cancers, and 92% and 82%, respectively, of tubular cancers. With regard to differentiation, a high proportion of cancers was reactive even for poorly-differentiated carcinoma, 81% with 2C3, 74% with 9B5, and 81% with 4A1, whereas fewer were reactive with 10B3 (49%) and 4D3 (56%).

There was no strict correlation of the reactivities of each MAb with any parameter examined (every MAb reacted with $>50\%$ of cancers in each subcategory). However, some trends did emerge from this analysis (Table II). MAb 4D3 reacted with mucinous and tubular carcinomas at a higher frequency (89 and 82%, respectively) than papillary carcinomas (56%); whereas anti-CEA antisera reacted with fewer mucinous carcinomas (79%) than papillary or tubular carcinomas (100 and 97%, respectively). MAb 4A1 showed a higher frequency of reactivity with expanding (91% positive) versus infiltrating carcinomas (73% positive). MAb 10B3 and 4D3 reacted with fewer carcinomas with a marked lymphocyte response (70 and 61%, respectively, c.f., 91% positive for each MAb in carcinoma with little lymphocytic response).

*Quantitation by ELISA of mucin antigens*

Sandwich ELISA were developed for the quantitation of mucins reactive with each of the MAb 2C3, 9B5, 10B3, 4A1 and 4D3 in the normal gastrointestinal tract and in cancers. An example of a standard curve for the one of the MAb, 4A1, with cancer mucin preparation no. 3266, is shown in Figure 6. This ELISA and similar ones using the other MAb could reliably detect 2–250 antibody-reactive units per ml. These assays were not affected by concentrations of guanidinium HCl $<0.4$ M and could therefore be used to assay tissue homogenates prepared in 4 M guanidinium HCl then diluted 1:10. Results of sandwich ELISA of tissue extracts from segments of the normal large intestine and from six specimens of colorectal cancer are shown in Figure 7. Mucins were subsequently purified from these tissue extracts by cesium chloride (CsCl) gradients, the yield of mucin antigenicity was 80 to 100%, thus accounting for virtually all of the tissue antigenicity.

**Table II** Correlation of mucin antigen detection with clinicopathological features of colorectal carcinoma

| *Clinico-pathological Feature* | *No. of cases* | *Percentage of cases positive for Ab reactivity* 2C3 | *9B5* | *10B3* | *4A1* | *4D3* | *CEA* |
|---|---|---|---|---|---|---|---|
| Stage | | | | | | | |
| A | 13 | 100 | 92 | 77 | 77 | 85 | 92 |
| B | 24 | 100 | 96 | 92 | 92 | 88 | 92 |
| C | 37 | 92 | 95 | 81 | 92 | 73 | 89 |
| D | 7 | 86 | 100 | 100 | 100 | 71 | 100 |
| Tumour size | | | | | | | |
| 0–10 $cm^3$ | 33 | 94 | 97 | 85 | 88 | 88 | 94 |
| 11–30 $cm^3$ | 31 | 97 | 90 | 87 | 90 | 77 | 90 |
| 30–150 $cm^3$ | 11 | 91 | 91 | 81 | 100 | 72 | 100 |
| Histological type | | | | | | | |
| Mucinous | 19 | 95 | 95 | 89 | 100 | 89 | 79 |
| Papillary | 9 | 89 | 100 | 78 | 78 | 56 | 100 |
| Tubular | 62 | 95 | 90 | 85 | 92 | 82 | 97 |
| Differentiation[a] | | | | | | | |
| Well | 43 | 88 | 83 | 86 | 79 | 74 | 79 |
| Mod-well | 10 | 70 | 80 | 70 | 50 | 60 | 80 |
| Mod | 88 | 92 | 86 | 70 | 79 | 72 | 80 |
| Mod-poor | 12 | 92 | 92 | 83 | 92 | 92 | 67 |
| Poor | 43 | 81 | 74 | 49 | 81 | 56 | 67 |
| Invasiveness | | | | | | | |
| Infiltrating | 26 | 96 | 100 | 88 | 73 | 85 | 96 |
| Expanding | 57 | 95 | 93 | 84 | 91 | 82 | 91 |
| Lymphocyte response | | | | | | | |
| Little | 11 | 91 | 91 | 91 | 100 | 91 | 82 |
| Moderate | 41 | 93 | 95 | 90 | 90 | 98 | 93 |
| Marked | 23 | 100 | 96 | 70 | 96 | 61 | 96 |
| Fibrosis | | | | | | | |
| Little | 26 | 96 | 88 | 85 | 92 | 69 | 88 |
| Moderate | 36 | 97 | 97 | 83 | 91 | 89 | 94 |
| Extensive | 23 | 91 | 96 | 91 | 100 | 83 | 96 |
| Site | | | | | | | |
| Rt. colon | 18 | 100 | 94 | 89 | 100 | 78 | 89 |
| Lt. colon | 18 | 100 | 100 | 78 | 89 | 72 | 83 |
| Rectum | 39 | 92 | 87 | 85 | 90 | 77 | 97 |
| Total | 100 | 95 | 90 | 85 | 91 | 77 | 92 |

[a]The differentiation sometimes varied from one part of a cancer to another, so for this parameter, correlation with mucin antigen reactivity was determined for each sample. Several samples were available from about half of the cases.

**Figure 6** Sandwich ELISA standard curve for anti-SIMA MAb 4A1 using cancer mucin preparation 3266.

(i) *Normal large intestine* The anti-LIMA MAb 2C3 and 9B5, as well as MAb 10B3, reacted strongly with mucin in all five segments of the large intestine from the ascending colon to the rectum; whereas the only trace or undetectable reactivity was evident using the anti-SIMA MAb 4A1 and 4D3 (Figure 7). Thus, for 2C3, the levels ranged from 6,000 to approximately 30,000 2C3-reactive units/g wet weight of tissue, with the highest levels generally found in the sigmoid colon and rectum (left side). As expected, 2C3-reactive LIMA was undetectable in extracts of normal duodenum, jejunum and ileum, $< 10$ units $g^{-1}$ tissue (data not shown). Mucin reactive with MAb 9B5 was detected at levels usually about 1,000 to 4,000 9B5-reactive units/g tissue (Figure 7), but only trace amounts were detected in the small intestine (data not shown). Mucin reactive with MAb 10B3 was also detected at levels ranging from 1,000 to 3,000 units/$g^{-1}$ tissue, and in this case, similar levels were also detected in the small intestine (data not shown).

In contrast to this, the anti-SIMA MAb 4A1 showed only traces of reactivity in the large intestine, ranging from 10 to 50 4A1-reactive units $g^{-1}$ tissue. The other anti-SIMA MAb 4D3 did not react with large intestinal mucin, i.e., $< 10$ 4D3-reactive units $g^{-1}$ tissue, compared with its strong reactivity with small intestinal mucin, 50,000 to 100,000 units $g^{-1}$ tissue (data not shown).

(ii) *Colorectal cancer specimens* In extracts of colorectal cancers, ELISA detected quantitative increases or decreases in the expression of MAb 2C3, 9B5 and 10B3-reactive mucins, which are normally detected in that organ, as well as the inappropriate expression of anti-SIMA 4D3 or 4A1-

**Figure 7** Quantitation by sandwich ELISA, using each of the five MAbs of mucin antigens from extracts of five segments of the normal large intestine and from six specimens of colorectal cancer. Different shading patterns are used to highlight different cancer specimens.

reactive mucins, not normally detected at significant levels in that organ (Figure 7). This analysis also demonstrated the heterogeneity of mucins in colorectal cancers. For example, cancer specimens 1, 2 and 4 show strong reactivity with all five MAb, mucin from specimen 3 reacts with 2C3, 10B3 and 4D3, specimen five with only 10B3 and 4D3, and specimen 6 with only 4D3 and 4A1.

Levels of 2C3-reactive mucin in colorectal cancer extracts, compared with levels in the normal colon extracts, were increased in two cases (25,000 and 250,000 units $^{-1}$), decreased in two cases (400 and 3,000 units $g^{-1}$), and not detected in two cases. Levels of 9B5-reactive mucin were elevated in one sample (20,000 units $g^{-1}$), within the normal range in two samples (3,300 and 3,9000 units $g^{-1}$), and not detected in three samples. The levels of 10B3-reactive mucin in the cancer samples were elevated in five to six cases, ranging from 11,000 to 400,000 units $g^{-1}$, and not detected in one case. Elevated levels of 4D3-reactive mucin were detected in all six cancer samples, ranging from 1,500 to 33,000 units $g^{-1}$. The levels of 4A1-reactive mucin in the cancer samples were elevated in four to six cases at 600 to 20,000 units $g^{-1}$, and not detected in two cases.

### Discussion

A new panel of MAb reactive with intestinal mucins was used to investigate the distribution and heterogeneity of antigen expression in 100 cases of colorectal carcinoma. The results were correlated with clinicopathological indices and compared with the distribution of a well characterised colorectal tumour antigen, CEA. The panel of MAb included two MAb, 4D3 and 4A1, that react with epitopes on SIMA, which is an oncofoetal antigen of the colon, but is not detectable in the normal adult colon (Hertzog *et al.*, 1991). The panel also included two MAb, 2C3 and 9B5, that recognised mucin normally localised predominantly in the normal large intestine and one MAb, 10B3, that reacted with epitopes on SIMA and LIMA.

From the combined data from immunohistochemistry of tissue sections and ELISA of mucins extracted from normal and cancer tissues, general conclusions can be made concerning the specificity of these MAb:

- Each MAb recognises a different epitope.
- In each case the epitope is present more than once on the antigen.
- Antigens extracted from normal and cancer tissues have buoyant densities characteristic for mucin glycoproteins.
- With the exception of the anti-SIMA MAb 4D3, the MAb recognise neuraminidase-insensitive epitopes.
- None of the MAb react with gastric mucin which distinguishes them from another series of gastrointestinal mucin antibodies (Bara *et al.*, 1984).
- The patterns of reactivity of these MAb, particularly in the normal gastrointestinal tract are different from that described for MAb to A, B, H and Lewis-type blood groups (Wolf *et al.*, 1989; Abe *et al.*, 1986; Itzkowitz *et al.*, 1986; Kim *et al.*, 1986; Sakomoto *et al.*, 1986; Schoentag *et al.*, 1987).
- The reactivity of the mucin antigens is resistant to formalin fixation and paraffin embedding of the tissues.
- The two anti-SIMA MAb recognise cancer mucin antigens that are abnormal for the colorectum.

As previously discussed, the sensitivity of the 2C3 and 4D3 epitopes to $\beta$-elimination, periodate oxidation or neuraminidase (for 4D3 only) suggest that these epitopes contain, or are influenced by carbohydrate moieties (Hertzog *et al.*, 1991). Further biochemical characterization of the epitopes for all of these MAbs is in progress, but is limited by the availability of native antigen in sufficient quantities. The reactivity of 9B5 or 4A1 with the Golgi zone is suggestive of possibly a peptide or core carbohydrate epitope; preliminary data suggest 4A1 reactivity is periodate sensitive (data unpublished) consistent with a carbohydrate-dependent epitope. Since the genes coding for two intestinal core proteins designated MUC2 and MUC3 have been cloned, (Gum *et al.*, 1989; 1990) it would be interesting to correlate these with SIMA and LIMA, and determine whether atypical SIMA expression in colorectal cancers is the result of altered glycosylation of normal core protein(s), or expression of new core protein(s).

Atypical reactivity of the anti-SIMA MAbs 4D3 and 4A1 in colorectal carcinomas was usually observed with tissue components associated with mucin, namely glandular lumen secretions, mucinous lakes and goblet cells. Thus, even the well differentiated goblet cells, when present in colorectal cancer, produce the abnormal antigen SIMA. In addition, undifferentiated carcinoma cells also contained material reactive with the two anti SIMA MAb, particularly 4A1, in which case staining was observed in the region of the apical membrane. The MAb 10B3, while reactive with both SIMA and LIMA in normal adults, showed a pattern of cellular reactivity in colorectal cancers that was similar to the anti-SIMA MAb, again highlighting the breakdown of normal mucin antigen patterns that occurs in colorectal cancer.

Abnormal SIMA expression was detected in a high proportion of colorectal carcinomas, even those at an early stage of development such as those at stages A and B (89% positive for 4D3, 86% positive for 4A1) and those of small size ($< 10\,cm^3$, 88% positive for 4D3 and 4A1). Thus aberrant SIMA expression may be an early event in colon carcinogenesis and is even present in the potentially premalignant perineoplastic mucosa (see below). The utility of the two anti-SIMA MAb and anti-CEA, all of which react with antigens not normally present in the colon, in the detection of neoplastic change in the colon and rectum, was further enhanced by the findings that these antibodies reacted with a high proportion of cancers, regardless of histological type, differentiation, stage, site, size, age, or sex.

However, despite the lack of an absolute association of mucin antigen reactivity with any single clinicopathological index, some trends did emerge. The anti-SIMA MAb 4D3 showed a tendency for stronger staining associated with a less malignant phenotype, namely carcinoma of stage A/B, smaller size, better differentiation. Both 4D3 and 10B3 positive tumours had a lower lymphocyte infiltrate, a feature previously reported for histologically-defined 'mucinous tumours' (Pihl, 1984) and interpreted as an indication that mucins may 'shield' cancers from the host immune response (Greaves *et al.*, 1980).

Over 90% of colorectal cancers contained mucin reactive with the two anti-LIMA MAb 2C3 and 9B5 in extracellular mucinous lakes or glandular lumen secretions as observed with the anti-SIMA MAb. However, in contrast to the cellular pattern of anti-SIMA reactivity, the two anti-LIMA MAb did not react with goblet cells in colorectal cancers (albeit the site of reactivity in the normal colon), but reacted with undifferentiated carcinoma cells, in the apical membrane region, and less frequently in the Golgi zone. Some undifferentiated carcinoma cells of the majority of colorectal cancers appear, therefore, to be capable of synthesising and secreting normal colonic mucin. Thus, the normal colonic mucin, LIMA is produced in colorectal cancers by atypical cells, possibly goblet cell precursors or intermediate cells (Dawson & Filipe, 1976) but not by goblet cells. In addition to this qualitative abnormality of LIMA production in colorectal cancers, quantitative differences from normal were detected, by ELISA using MAb 2C3 or 9B5, as increased production in some cases and absence in others. The occurrence of LIMA in the cancers showed no trends with any clinicopathological parameters; the anti-LIMA MAb and the anti-SIMA MAb even reacted with a high proportion of colorectal cancers that would have been diagnosed histologically as 'non-mucinous'. These observations may be explained by the reactivity of these MAb frequently with mucin antigens in cellular or tissue compartments not normally associated with mucins.

The atypical subcellular and extracellular distribution of reactivity with these anti-mucin MAb is a reflection of altered glycoprotein biosynthesis and warrants further investigation at the ultrastructural and biochemical level. Altered metabolism may take the form of incomplete synthesis, degradation or synthesis of new (neo-) molecules as proposed for other differentiation-associated glycoconjugates (Hakomori & Kannagi, 1983). However, mucins differ from other tumour marker antigens, such as those on the cell surface, because they are more extensively glycosylated and are naturally secreted molecules. Mucin production by tumours may therefore be more readily detected in biological fluids, and may have different consequences for tumour progression. Abnormal mucin production may influence the progression of the tumour by affecting processes such as growth or invasion, or by presenting 'unseen' antigens to the host immune system. Furthermore, abnormal mucin glycoprotein metabolism or distribution may be an important factor in determining whether these antigens reach the blood, as proposed for CEA (Hamada *et al.*, 1985). Indeed, each of these MAb, on occasions, was observed to stain serum in blood vessels in specimens of colorectal cancer, and preliminary studies using ELISA have also detected mucin antigens in blood samples from some patients with colorectal cancer.

Whatever the underlying cause of the altered mucin glycoprotein metabolism in colorectal cancers, it is interesting that it varies in detail from one cancer to the next. Thus, cancers showed different profiles of reactivity with the individual anti-mucin MAb and CEA, evident in both immunohistochemical and ELISA studies. The reactivity of different regions of a cancer specimen were also frequently complementary in nature. Similar 'mosaicism' of reactivity in cancers has been described for another series of related tumour antigens (Nakasaki *et al.*, 1989). A cocktail of complementary antibodies would therefore react with a higher proportion of cancers and with more cells in a tumour than any of these individual antibodies, and may be the optimal reagent to use for tissue or blood diagnosis, immunolocalisation or tumours, or delivery of immunotoxins.

In the perineoplastic mucosa, the frequency of reactivity with the anti-SIMA MAbs 4D3 and 4A1 decreased only slightly from TM-I to TM-II (70 and 90% to 62 and 86% respectively), compared with the dramatic drop in frequency and intensity of reactivity with CEA (65% in TM-I, and 35% in TM-II). Similarly, abnormal mucin antigen expression detected by one of the anti-SIMA MAbs 4D3 or 4A1 was detected in the morphologically normal mucosa in a high proportion of cases (80%) versus CEA (24%). Clearly, these apparently normal cells are undergoing biochemical change. It remains an open question as to whether this change is precancerous (Filipe, 1969; Decaens *et al.*, 1983) or reactive (Isaacson & Attwood, 1979; Boland & Kim, 1987). Future studies will be directed to the factors that may be responsible for such changes, and the extent of these or similar changes in mucosa distant from the tumour.

In conclusion, we have described alterations of mucin production in the majority of colorectal cancers of every stage, differentiation and histological type, size and site. Not only were the new, atypical mucins (SIMA) produced, but the normal colonic mucin (LIMA) was found in abnormal cell types, subcellular and tissue compartments. The broad reactivity of this panel of MAb would be an advantage for their use, perhaps in combination with each other and/or CEA, in immunolocalisation, serological monitoring, or therapy of colorectal cancer.

### References

ABE, K., HAKOMORI, S. & OHSHIBA, S. (1986). Differential expression of difucosyl type 2 chain (Le-y) defined by monoclonal antibody AH6 in different locations of colonic epithelia, various histological types of colonic polyps, and adenocarcinomas. *Cancer Res.*, **46**, 2639.

ALLEN, A. (1983). Mucus – a protective secretion of complexity. *TIBS*, **5**, 169.

BARA, J., LOISILLIER, F. & BURTIN, P. (1980). Antigens of gastric and intestinal mucous cells in human colonic tumours. *Br. J. Cancer*, **41**, 209.

BARA, J., NARDELLI, J., GADENNE, C., PRADE, M. & BURTIN, P. (1984). Differences in the expression of mucus-associated antigens between proximal and distal human colon adenocarcinomas. *Br. J. Cancer*, **49**, 495.

BHATTACHARYA, M., CHATTERJEE, S.K., BARLOW, J.J. & FUJI, H. (1982). Monoclonal antibodies recognizing tumour-associated antigen of human ovarian mucinous cystadenocarcinomas. *Cancer Res.*, **42**, 1650.

BOLAND, C.R. & KIM, Y.S. (1987). Transitional mucosa of the colon and tumour growth factors. *Med. Hypotheses*, **22**, 237.

BRADFORD, M.M. (1976). A rapid and sensitive method for the quantitation of microgram quantities of protein utilizing the principle of protein-dye binding. *Anal Biochem.*, **72**, 248.

DAWSON, P.A. & FILIPE, M.I. (1976). An ultrastructural application of silver methenamine to the study of mucin changes in the colonic mucosa adjacent to and remote from carcinoma. *Histochem. J.*, **8**, 143.

DECAENS, C., BARA, J., ROSA, B., DAHER, N. & BURTIN, P. (1983). Early oncofetal antigenic modifications during rat colonic carcinogenesis. *Cancer Res.*, **43**, 355.

FEIZI, T. & CHILDS, R.A. (1985). Carbohydrate structures of glycoproteins and glycolipids as differentiation antigens, tumour-associated antigens, and components of receptor systems. *TIBS*, **1**, 24.

FILIPE, M.I. (1969). Value of histochemical reactions for mucosubstances in the diagnosis of certain pathological conditions of the colon and rectum. *Gut*, **10**, 577.

GOLD, D.V. & MILLER, F. (1978). Comparison of human mucoprotein antigen from normal and neoplastic mucosa. *Cancer Res.*, **38**, 3204.

GREAVES, P., FILIPE, M.I. & BRANFOOT, B.M. (1980). Transitional mucosa and survival in colorectal cancer. *Cancer*, **46**, 764.

GRIFFITHS, A.B., BURCHELL, J., GENDLER, S. & 4 others (1987). Immunological analysis of mucin molecules expressed by normal and malignant mammary epithelial cells. *Int. J. Cancer*, **46**, 319.

GUM, J.R., BYRD, J.C., HICKS, J.W., TORIBARA, N.W., LAMPORT, D.T.A. & KIM, Y.S. (1989). Molecular cloning of human intestinal mucin CDNAs. Sequence analysis and evidence for genetic polymorphism. *J. Biol. Chem.*, **264**, 6480.

GUM, J.R., HICKS, J.W., SWALLOW, D.M. & 5 others (1990). Molecular cloning of CDNAs derived from a novel human intestinal mucin gene. *Biochem. Biophys. Res. Comm.*, **171**, 407.

HAKOMORI, S. & KANNAGI, R. (1983). Glycosphingolipids as tumour-associated and differentiation markers. *J. Natl Cancer Inst.*, **71**, 231.

HAMADA, Y., YAMAMURA, M., HIOKI, K., YAMAMOTO, M., NAGURA, H. & WATANABE, K. (1985). Immunohistochemical study of carcinoembryonic antigen in patients with colorectal cancer. Correlation with plasma carcinoembryonic antigen levels. *Cancer*, **55**, 136.

HERTZOG, P.J., MA, J., ROBINSON, H.C., MACKAY, I.R. & LINNANE, A.W. (1991). Oncodevelopmental expression of the human intestinal mucin glycoprotein antigens in gastrointestinal epithelium defined by monoclonal antibodies. *Int. J. Cancer*, **48**, 355.

ISAACSON, P. & ATTWOOD, P.R.A. (1979). Failure to demonstrate specificity of the morphological and histochemical changes in mucosa adjacent to colonic carcinoma (transitional mucosa). *J. Clin. Pathol.*, **32**, 214.

ITZKOWITZ, S.H., YUAN, M., FUKUSHI, Y. & 6 others (1986). Lewis-x- and sialylated Lewis-x-related antigen expression in human malignant and nonmalignant colonic tissues. *Cancer Res.*, **46**, 2627.

JASS, J.R., ATKIN, W.S., CUZICK, J. & 4 others (1986). The grading of colorectal cancer: historical perspectives and a multivariate analysis of 447 cases. *Histopathology*, **10**, 437.

KIM, Y.S., YUAN, M., ITZKOWITZ, S.H. & 5 others (1986). Expression of Le-y and extended Le-y blood group-related antigens in human malignant, premalignant, and nonmalignant colonic tissues. *Cancer Res.*, **46**, 5985.

MA, J., DE BOER, W.G.R.M., WARD, H.A. & NAIRN, R.C. (1980). Another oncofetal antigen in colonic carcinoma. *Br. J. Cancer*, **41**, 325.

NAKASAKI, H., MITOMI, T., NOTO, T. & 6 others (1989). Mosaicism in the expression of tumour-associated carbohydrate antigens in human colonic and gastric cancers. *Cancer Res.*, **49**, 3662.

PIHL, E. (1984). Prognostic and clinical significance of mucin production in colorectal tumours. *Int. Med. Specialist*, **5**, 39.

SAKAMOTO, J., FURUKAWA, K., CORDON-CARDO, C. & 5 others (1986). Expression of Lewis-a, Lewis-b, X and Y blood group antigens in human colonic tumours and normal tissue and in human tumour-derived cell lines. *Cancer Res.*, **46**, 1553.

SCHOENTAG, R., PRIMUS, F.J. & KUHNS, W. (1987). ABH and Lewis blood group expression in colorectal carcinoma. *Cancer Res.*, **47**, 1695.

WOLF, B.C., SALEM, R.R., SEARS, H.F. & 6 others (1989). The expression of colorectal carcinoma-associated antigens in the normal colonic mucosa. An immunohistochemical analysis of regional distribution. *Am. J. Pathol.*, **135**, 111.